# 20-HETE and EETs in Diabetic Nephropathy: A Novel Mechanistic Pathway

**DOI:** 10.1371/journal.pone.0070029

**Published:** 2013-08-02

**Authors:** Stephanie Eid, Rita Maalouf, Ayad A. Jaffa, Joseph Nassif, Ahmed Hamdy, Awad Rashid, Fuad N. Ziyadeh, Assaad A. Eid

**Affiliations:** 1 Department of Anatomy, Cell Biology and Physiology, Faculty of Medicine, American University of Beirut, Beirut, Lebanon; 2 Department of Sciences, Faculty of Natural and Applied Sciences, Notre Dame University, Zouk, Lebanon; 3 Department of Biochemistry and Molecular Genetics, Faculty of Medicine, American University of Beirut, Beirut, Lebanon; 4 Department of Obstetrics and Gynecology, Faculty of Medicine, American University of Beirut, Beirut, Lebanon; 5 Department of Nephrology, Hamad Medical Corporation, Doha, Qatar; 6 Department of Internal Medicine, Faculty of Medicine, American University of Beirut, Beirut, Lebanon; University of Florida, United States of America

## Abstract

Diabetic nephropathy (DN), a major complication of diabetes, is characterized by hypertrophy, extracellular matrix accumulation, fibrosis and proteinuria leading to loss of renal function. Hypertrophy is a major factor inducing proximal tubular epithelial cells injury. However, the mechanisms leading to tubular injury is not well defined. In our study, we show that exposure of rats proximal tubular epithelial cells to high glucose (HG) resulted in increased extracellular matrix accumulation and hypertrophy. HG treatment increased ROS production and was associated with alteration in CYPs 4A and 2C11 expression concomitant with alteration in 20-HETE and EETs formation. HG-induced tubular injury were blocked by HET0016, an inhibitor of CYPs 4A. In contrast, inhibition of EETs promoted the effects of HG on cultured proximal tubular cells. Our results also show that alteration in CYPs 4A and 2C expression and 20HETE and EETs formation regulates the activation of the mTOR/p70S6Kinase pathway, known to play a major role in the development of DN. In conclusion, we show that hyperglycemia in diabetes has a significant effect on the expression of Arachidonic Acid (AA)-metabolizing CYPs, manifested by increased AA metabolism, and might thus alter kidney function through alteration of type and amount of AA metabolites.

## Introduction

Diabetic nephropathy (DN) is a major risk factor for cardiovascular morbidity and mortality. DN is characterized by glomerular, vascular, tubular, and interstitial lesions that initially develop in the absence of measurable dysfunction [1]. Although DN was traditionally considered to be primarily a glomerular disease, it is now widely accepted that the rate of deterioration of kidney function correlates best with the degree of tubulointerstitial fibrosis [1]. One of the earliest renal pathological changes in diabetes is an increase in tubular basement membrane mass that accompanies the development of renal hypertrophy [2]. Diabetic kidney disease is characterized by progressive accumulation of extracellular matrix proteins, including fibronectin, collagen or laminin in the tubular compartment. Tubulointerstitial fibrosis is likely to represent a final common pathway leading to end-stage renal disease and the need for dialysis or transplantation [1]–[4]. Although the etiology of the tubulointerstitial pathology in DN is not fully understood, much attention has focused on the role of high glucose (HG) *per se*. Tubular cells are a primary target of hyperglycemia and chronic exposure to elevated blood glucose levels contributes to the tubulointerstitial changes seen in overt DN [1]–[3]. While the role of diabetes in increased generation of extracellular matrix protein is well established, the mechanisms by which hyperglycemia/glucose results in the accumulation of these proteins are unclear.

Arachidonic acid (AA) is metabolized by several cytochrome 450 (CYP) isoforms to produce 20-hydroxyeicosatetraenoic acid (20-HETE) and epoxyeicosatrienoic acids (EETs). CYPs of the 4A and 4F subfamilies are highly expressed in the kidneys and form 20-HETE [5]–[9]. CYPs of the 2B, 2C and 2J subfamilies form EETs. Data have shown that CYPs 2C and 2J are highly expressed in the proximal tubules of the kidneys [10], [11].

Beside the involvement of 20-HETEs and EETs in hypertension [12]–[16], little is known about their involvement in other physical and physiological changes wrought by diabetes in the kidney. The regulation of CYPs 450 is tissue and disease specific. Recent studies have implicated alterations in CYPs 450 metabolites as contributing to renal damage in obesity and diabetes [17]–[22]. Also, it has been shown that 20-HETE and EETs have multiple and opposing functions depending on the location of their production and target cells/tissues [22]–[25]. The mechanism by which glucose alters CYPs expression, activity and their metabolites production is still to be elucidated. More importantly the role exerted by these CYPs and their metabolites on proximal tubular cells integrity and function are not known.

Oxidative stress is thought to be a critical factor in the development of DN. ROS has been shown to play a major role in diabetes and its complication, i.e. DN [26]–[28]. CYPs have been shown to be significant sources of oxidative stress in kidneys and other organs, i.e. coronary arteries and liver [22], [29]–[31]. Alternatively, these CYPs or the products that they form might also be sensitive to the presence of ROS produced by other sources [31]. Nonetheless, the involvement of CYPs family and their metabolites in the proximal tubular cells to produce significant ROS leading to nephropathy and their crosstalk with other signaling pathways involved in diabetic kidney injury remains to be elucidated.

In this study, we demonstrate that HG induces ROS production, matrix protein accumulation and hypertrophy in cultured rat proximal tubular epithelial cells. These effects are mediated through the alteration of CYPs 4A and 2C protein expression and alteration in their metabolites production 20-HETE and EETs. We also provide the first evidence that alteration in CYPs 4A and 2C activity and metabolites formation regulates the activation of the mTOR/p70S6Kinase pathway known to play a pivotal role in the development of DN.

## Research Design and Methods

### Cell Culture

Rat proximal tubular epithelial cells (Rat PTCs) [32], were maintained in Dulbecco’s modified Eagle’s medium containing 5 mM glucose (DMEM) plus 10% fetal bovine serum in a 5% CO2 atmosphere at 37°C. Near confluent cells are grown in DMEM containing 0.1% FBS and 5 mmol/l D-glucose (normal glucose; NG) for 18 to 24 h before the experiment. Cells are then incubated for 1 h with *N*-hydroxy-*N*-(4-butyl-2-methylphenol) formamidine (HET0016; 1.0 µmol/l, 1 h) [22], [33], or with N-methylsulfonyl-6-(2-propargyloxyphenyl) hexanamide (MSPPOH; 5 µmol/l, 1 h) [34], followed by the addition of HG for the various time periods.

### Detection of Intracellular ROS

The peroxide-sensitive fluorescent probe 2′,7′-dichlorodihydrofluorescin diacetate (Molecular Probes) is used to measure intracellular ROS as previously described [22], [35]. Cells are grown in 12 or 24 well plates and serum-deprived for 24 h. Immediately before the experiments, cells are washed with HBSS (Hanks Buffer Salt Solution) containing Ca^++^ and Mg^++^ and loaded with 50 µM 2′,7′-dichlorodihydrofluorescin diacetate dissolved in HBSS for 30 min at 37°C. Cells are then incubated for 1 hour with 1.0 µmol/l of HET0016, a potent inhibitor of CYP4A (20-HETE biosynthesis) or 5 µmol/l of MSPPOH, a selective inhibitor of microsomal CYPs450 epoxidase activity (EETS and DiHETE biosynthesis), followed by the addition of the agonist or vehicle for various time periods. DCF fluorescence is detected at excitation and emission wavelengths of 488 and 520 nm, respectively, and measured in a multiwell fluorescence plate reader (Spectramax Gemini EM, Molecular Devices).

### Microsomes Isolation

Rats proximal tubular epithelial cells in serum-free medium were homogenized in a 10 mmol/l potassium phosphate buffer (pH 7.7) containing 250 mmol/l sucrose, 1 mmol/l EDTA, 10 mmol/l magnesium chloride, 2 µmol/l leupeptin, 1 µmol/l pepstatin, 2 µg/ml aprotinin, and 0.1 µmol/l phenylmethylsulfonyl fluoride. Microsomes are prepared by differential centrifugation as described [22], [36] and used for western blotting.

### Western Blotting Analysis

Cultured rats proximal tubular epithelial cells are grown to near confluency in 60 or 100 mm dishes and serum-deprived for 24 h. All incubations are carried out in serum-free DMEM containing 0.1% FBS at 37°C for short duration (3 h and 6 h) and for long duration (24 h and 48 h). The cells are lysed in radioimmune precipitation buffer (20 mM Tris·HCl, pH 7.5, 150 mM NaCl, 5 mM EDTA, 1 mM Na_3_VO_4_, 1 mM phenylmethylsulfonyl fluoride, 20 µg/ml aprotinin, 20 µg/ml leupeptin,1% NP-40) at 4°C for 30 min. The cell lysates are centrifuged at 10,000 *g* for 30 min at 4°C. Protein in the supernatants is measured using the Bio-Rad method. For immunoblotting, proteins (40–80 µg) are separated on 12.5% SDS-PAGE and transferred to polyvinylidene difluoride membranes [22], [35]. Blots are incubated with rabbit polyclonal anti-CYP4A (1∶500, Abcam), rabbit polyclonal anti-CYP2C11 (1∶1000, Abcam), rabbit polyclonal anti-fibronectin (1∶1000, Abcam), rabbit polyclonal anti-Collagen (1∶1000, Abcam), rabbit polyclonal anti-phospho-mTOR^Ser2448^ (1∶1000, Abcam), rabbit polyclonal anti-mTOR (1∶1000, Abcam), rabbit polyclonal anti-phospho-p70 S6 Kinase^Thr389^ (1∶1000, Abcam), rabbit polyclonal anti-p70 S6 Kinase (1∶1000, Abcam). The primary antibodies are detected using horseradish peroxidase-conjugated IgG (1∶2500 or 1∶5000). Bands are visualized by enhanced chemiluminescence. Densitometric analysis is performed using National Institutes of Health Image software.

### 20-HETE Formation

Levels of 20-HETE are measured using the 20-HETE Elisa kit (Detroit R&D, INC., USA) according to the manufacturer protocol. This competitive ELISA kit determines the levels of 20-HETE in biological samples.

### EETs Formation

Cytochrome P4502C11 is a predominantly found in rats kidney and it produces mainly 14,15-EETs. Levels of 14,15-EETs are measured using the 14,15-EET/DHET Elisa kit (Detroit R&D, INC., USA) according to the manufacturer protocol.

### Hypertrophy Assays

Rat proximal tubular cells in 12-well plates are starved in serum-free medium and incubated with medium containing 5 mmol/l glucose (NG) or with 25 mmol/l glucose (HG) for 6 h or 48 h in the presence or absence of HET0016 or MSPPOH. In the last 2 h, the cells are labeled with 1 µCi/ml of [^3^H]-thymidine and [^35^S]-methionine. The washed cells are fixed in cold 10% trichloroacetic acid, and the precipitates are dissolved in 0.25 mol/l NaOH containing 0.1% SDS and then counted as previously described [37], [38]. Using this assay, there was no increase in DNA synthesis in response to HG. Therefore, increase in protein synthesis was used as a surrogate for hypertrophy [38].

### Statistical Analysis

Results are expressed as mean ± S.E. Statistical significance was assessed by Student’s unpaired t test and determined as probability (P) ≤0.05.

## Results

### High Glucose Induces Oxidative Stress, Stimulates Matrix Protein Accumulation, and Induces Protein Synthesis in Proximal Tubular Epithelial Cells

**Figure 1:**
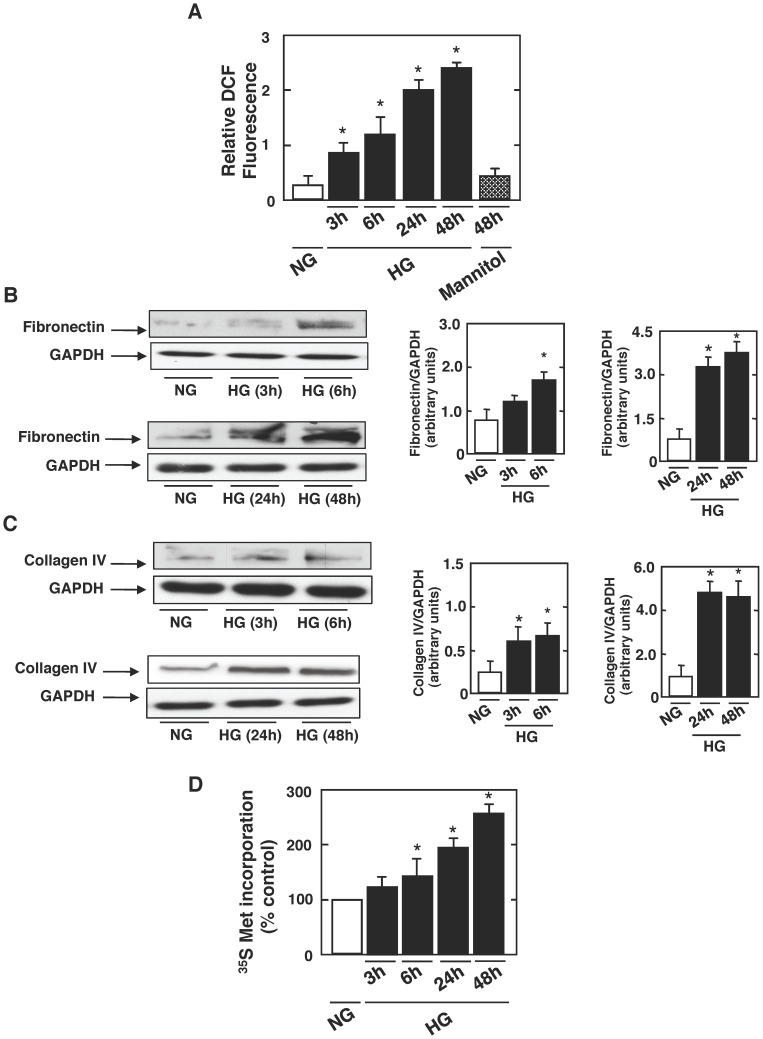
Temporal effect of high glucose on ROS generation in rat proximal tubular cells. Rat proximal tubular cells were exposed to either high glucose (HG; 25 mmol/l) or normal glucose (NG; 5 mmol/l) for the indicated time periods. ***A***: ROS generation was measured by DCF with a multiwall fluorescence plate reader as described in research design and methods. Values are the means ± SE of three independent experiments (*n = *3). *P<0.05, high glucose vs. control. ***B*** *and* ***C***: Representative western blots showing the expression of fibronectin and collagen type IV protein in rat proximal tubular epithelial cells. GAPDH was included as a control for loading and the specificity of change in protein expression. The histograms show quantitation of the results from three independent experiments (*n = *3). *P<0.05, high glucose vs. control. ***D***: determination of [35S]-methionine incorporation; the means of triplicate measurements are shown. Values are the means ± SE of three independent experiments (*n = *3). *P<0.05, high glucose vs. control.

Exposure of rat proximal tubular epithelial cells to 25 mmol/l glucose (high glucose; HG) results in a relatively rapid generation of ROS, as measured by DCF fluorescence, when compared to cells incubated in 5 mmol/l glucose (normal glucose; NG) or mannitol that is used as osmotic control. ROS generation was detected after 3 h of exposure to HG and was sustained up to 48 h (Fig. 1*A*). As expected, mannitol didn’t have any effect on ROS production (Fig. 1*A*). Consistent with that, exposure of the cells to HG resulted in tubulointerstitial changes (matrix accumulation) and proximal tubular cells hypertrophy (Figs. 1*B*–1*D*); HG treatment stimulates matrix protein accumulation, assessed by increased fibronectin expression at 6 h, an effect sustained up to 48 h (Fig. 1*B*) and increased collagen IV expression starting at 3 h of HG exposure and sustained up to 48 h (Fig. 1*C*). Also, HG significantly increased *De novo* protein synthesis measured by [^35^S] methionine incorporation (Fig. 1*D*), a measure of cell hypertrophy. This effect was slightly but significantly observed at 6 h and sustained up to 48 h of incubation with HG concentration (Fig. 1*D*). In the rest of the experiments, rat PTCs will be incubated for 6 h with HG concentration (short-term exposure) or 48 h (long-term exposure), since the desired effect was seen at these 2 time points.

### HG Alters CYP4A, CYP2C11 Proteins Expression, and Arachidonic Acid Metabolites 20-HETE and 14,15-EETs Formation

**Figure 2:**
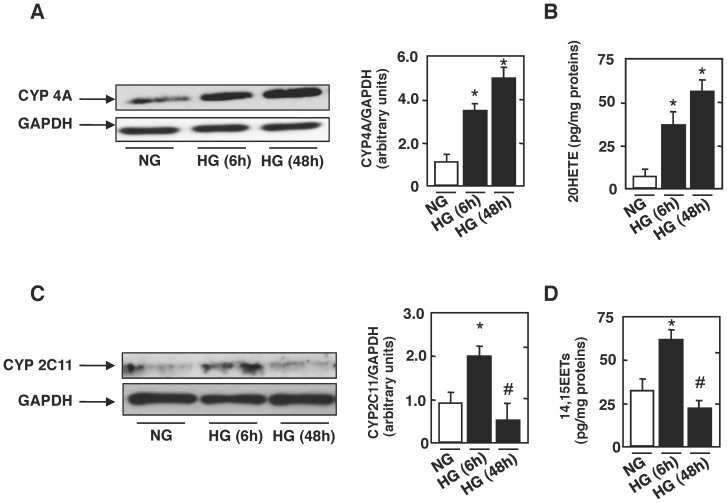
High glucose alters CYP4A and CYP2C11 protein expression, and arachidonic acid metabolites 20-HETE and 14,15-EETs production. Rat proximal tubular cells were exposed to either high glucose (HG; 25 mmol/l) or normal glucose (NG; 5 mmol/l) for the indicated time periods. ***A***: Microsomes were isolated from rat proximal tubular epithelial cells. Representative western blots showing the expression of CYP4A. GAPDH was included as a control for loading and the specificity of change in protein expression. The histograms show quantitation of the results from three independent experiments (*n = *3). *P<0.05, high glucose vs. control. ***B*** *:* Histograms representing 20-HETE formation measured in rat proximal tubular epithelial cells using 20-HETE ELISA kit. The histograms show quantitation of the results from three independent experiments (*n = *3). *P<0.05, high glucose vs. control. ***C***: Microsomes were isolated from rat proximal tubular epithelial cells. Representative western blots showing the expression of CYP2C11. GAPDH was included as a control for loading and the specificity of change in protein expression. The histograms show quantitation of the results from three independent experiments (*n = *3). *P<0.05, high glucose vs. control. ***D*** *:* Histograms representing 14,15-EETs formation measured in rat proximal tubular epithelial cells using 14,15-EETS/DiHETE ELISA kit. The histograms show quantitation of the results from three independent experiments (*n = *3). *P<0.05, high glucose vs. control.

We next examined whether CYP4A contributes to HG induced-rat proximal tubular epithelial cells injury. Microsomes were isolated from cells incubated in NG or HG. Our data show that CYP4A protein expression was increased in microsomes isolated from cells incubated in HG for 6 h or 48 h compared to cells incubated in NG (Fig. 2*A*). The increase of CYP4A expression was accompanied by an increase in 20-HETE formation (Fig. 2*B*). In parallel, we assessed the expression of CYP2C11, a major cytochrome present in rats’ kidney [39], and measured the formation of 14,15-EETs, major biologically active metabolite produced by CYP2C [39]. Our data show a significant increase in CYP2C11 protein expression at 6 h paralleled by a significant increase in 14,15-EETs formation (Fig. 2*C–*2*D*). Interestingly, when cells are exposed to HG for 48 h (long term exposure), we see a decrease in CYP2C11 protein expression and 14,15-EETs formation when compared to cells incubated in HG for 6 h period (Fig. 2*C–*2*D*). More importantly, when cells were incubated with HG for 48 h, CYP2C11 protein expression and 14,15 EETs formation were slightly but not significantly decreased as compared to cells exposed to NG (Fig. 2*C–*2*D*).

### 20-HETE Generated by CYP4A Mediates the Effect of HG on Tubulointerstitial Changes and Tubular Injury

**Figure 3:**
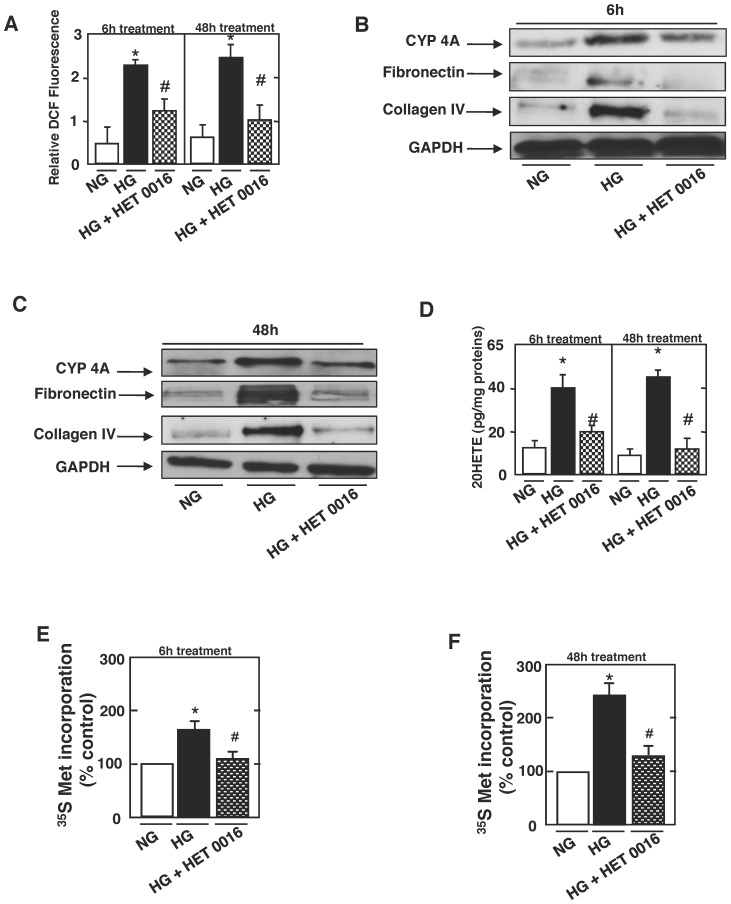
20-HETE generated by CYP4A mediates the effect of high glucose on tubulointerstitial changes and tubular injury. Rat proximal tubular epithelial cells were exposed to high glucose for 6 h or 48 h, in the absence or presence of *N*-hydroxy-*N*-(4-butyl-2-methylphenol) formamidine (HET0016; 1.0 µmol/l, 1 h). ***A***: ROS generation was measured by DCF with a multiwall fluorescence plate reader as described in research design and methods. Values are the means ± SE of three independent experiments (*n = *3). *P<0.05 versus control; ^#^P<0.05 versus HG. ***B*** and ***C***: Representative western blots showing the expression of CYP4A (in microsomes isolated from cells), fibronectin and collagen type IV. GAPDH was included as a control for loading and the specificity of change in protein expression. ***D***: Histograms representing 20-HETE formation measured in rat proximal tubular epithelial cells using 20-HETE ELISA kit. The histograms show quantitation of the results from three independent experiments (*n = *3). *P<0.05 versus control; ^#^P<0.05 versus HG. ***E*** and ***F***: determination of [35S]-methionine incorporation; the means of triplicate measurements are shown. *P<0.05 versus control; ^#^P<0.05 versus HG.

We next determined whether CYP4A-derived 20-HETE mediates the effect of HG on ROS generation, matrix protein accumulation and hypertrophy in rat proximal tubular cells. DCF fluorescence was used to measure ROS in cells exposed to HG, in the absence or presence of HET0016. The increase in ROS generation induced by HG was inhibited by HET0016 treatment at 6 h and 48 h (Fig. 3*A*). These data indicate that 20-HETE mediated the effect of HG on ROS production. We next show that the redox pathway implicating CYP4A-dependent generation of 20-HETE mediates the effect of HG-induced tubulointerstitial changes and tubular hypertrophy. Data in Fig. 3*B and 3C* show that HG induced CYP4A protein expression and 20-HETE formation was attenuated by HET0016 treatment at 6 h and 48 h of HG exposure. In parallel, HET0016 reverse the effect of HG-induced fibronectin and collagen IV protein expression (Fig. 3*B and 3C*) and inhibits hypertrophy (Fig. 3*E*). Our findings indicate that the increased CYP4A expression/20-HETE formation induce ROS generation and result in tubulointerstitial changes and tubular hypertrophy.

### Alteration in EETs Production by CYP2C Mediate the Effect of HG on Tubulointerstitial Changes and Tubular Injury

**Figure 4:**
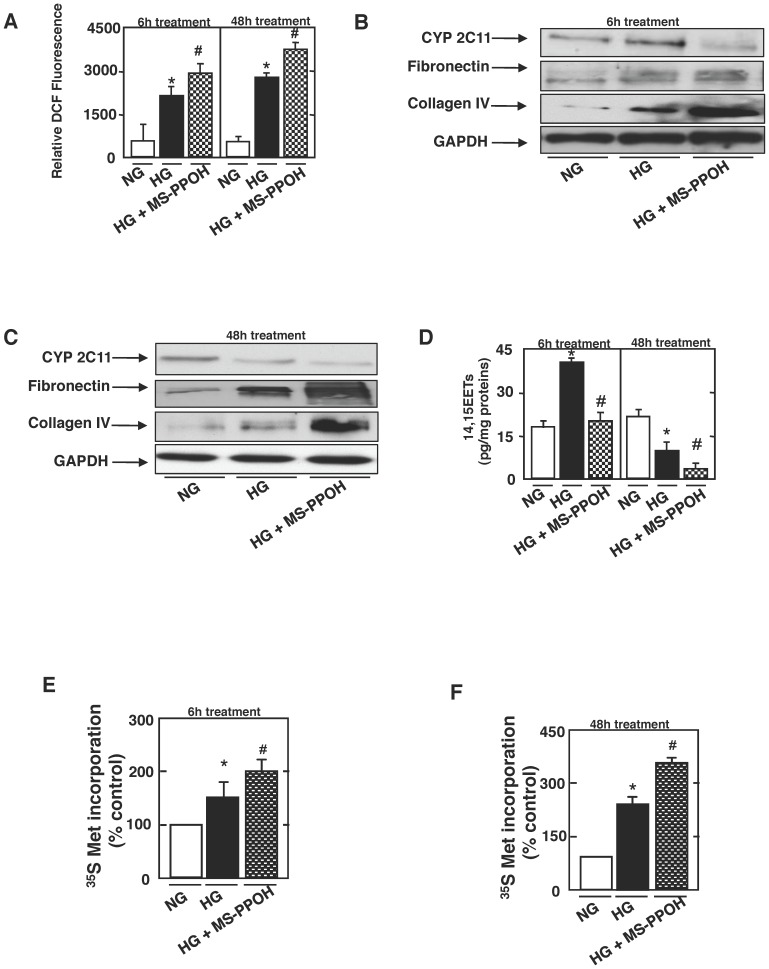
Alteration in EETs production by CYP2C mediate the effect of high glucose on tubulointerstitial changes and tubular injury. Rat proximal tubular epithelial cells were exposed to high glucose for 6 h or 48 h, in the absence or presence of N-methylsulfonyl-6-(2-propargyloxyphenyl) hexanamide (MSPPOH; 5 µmol/l, 1 h). ***A***: ROS generation was measured by DCF with a multiwall fluorescence plate reader as described in research design and methods. Values are the means ± SE of three independent experiments (*n = *3). *P<0.05 versus control; ^#^P<0.05 versus HG. ***B*** and ***C***: Representative western blots showing the expression of CYP42C11 (in microsomes isolated from cells), fibronectin and collagen type IV. GAPDH was included as a control for loading and the specificity of change in protein expression. ***D***: Histograms representing 14,15-EETs formations measured in rat proximal tubular epithelial cells using 14,15-EETs/DiHETE Elisa kit. The histograms show quantitation of the results from three independent experiments (*n = *3). *P<0.05 versus control; ^#^P<0.05 versus HG. ***E*** and ***F***: determination of [35S]-methionine incorporation; the means ± SE of triplicate measurements are shown. *P<0.05 versus control; ^#^P<0.05 versus HG.

A protective role of EETs has been described in vascular cells. However, the role played by EETs in diabetic kidney injury is under investigation. We examined whether alteration in CYP2C-derived EETs formation mediates the effect of HG on ROS generation, matrix accumulation and increased hypertrophy in rats proximal tubular cells. ROS generation was assessed in cells exposed to HG, in the absence or presence of MSPPOH. Interestingly, we show that pretreatment of cells with MSPPOH shows an increased stimulation of ROS production when compared to cells treated with HG (Fig. 4*A*). We also show that the signaling pathway implicating CYP2C11-dependent formation of EETs mediates the effect of HG-inducing tubulointerstitial changes and tubular hypertrophy. Our data in Fig. 4*B* show that short-term exposure to HG (6 h) induces CYP2C11 protein expression as well as 14,15-EETs formation, an effect that is inhibited when cells are pretreated with MSPPOH (Fig. 4*B and 4D*). In contrast, when cells are exposed to HG for longer periods (48 h), CYP2C11 protein expression as well as 14,15-EETs formation were significantly decreased (Fig. 4*C and 4D*). In parallel experiments, rat proximal tubular cells exposed to HG in the presence or absence of MSPPOH, show a significative induction of fibronectin and collagen IV protein expression and increased stimulation of cellular hypertrophy when compared to cells exposed to HG (Fig. 4*C* and Figs. 4*H*–4*J*). These findings indicate that the decrease in CYP2C expression/EETs formation lead to an increase of ROS production and worsen proximal tubular epithelial cells injury.

### CYP4A and CYP2C Regulate HG-induced Proximal Tubular Epithelial Cells Injury Through an mTOR Dependent Mechanism

**Figure 5:**
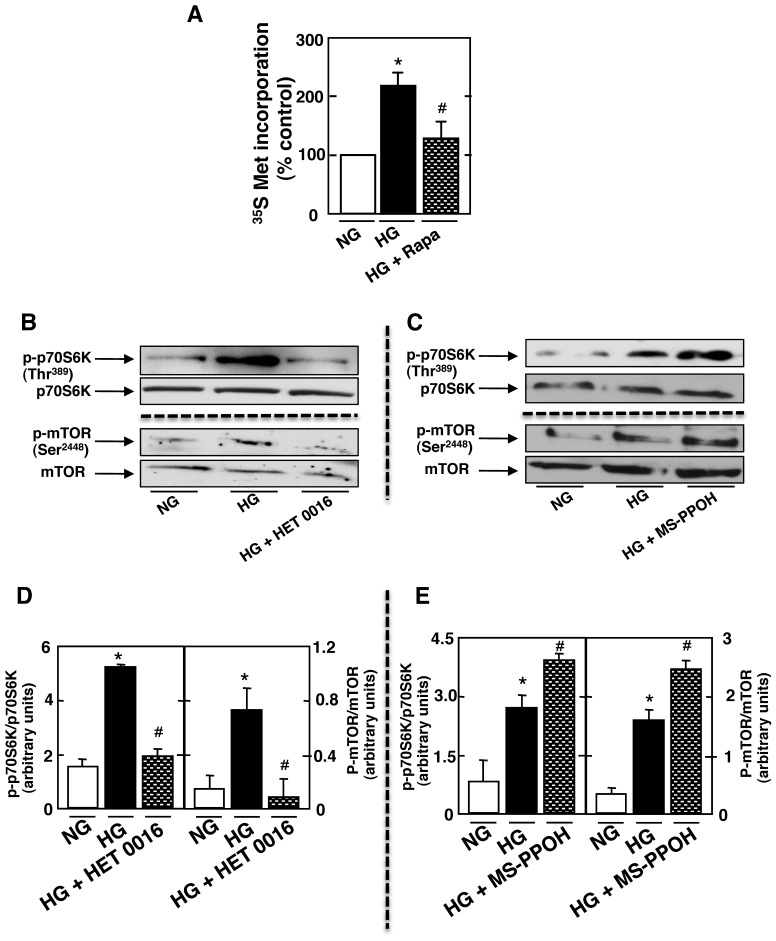
CYP4A and CYP2C regulate HG-induced proximal tubular epithelial cells injury through an mTOR dependent mechanism. Rat proximal tublar epithelial cells were exposed to 25 mmol/l glucose (HG) with or without rapamycin (10 nanomol/l; 1h) for 48 h. ***A***: Determination of [35S]-methionine incorporation; the means of triplicate measurements are shown. *P<0.05 versus control; ^#^P<0.05 versus HG. ***B*** and ***C***: Representative Western blot of p-p70S6K^Thr389^, p70S6K, p-mTOR^Ser2448^, mTOR treated with HG in the presence or absence of 10 nm rapamycin. ***D*** *and* ***E***: Histograms show quantitation of the results from three independent experiments (*n = *3). *P<0.05 versus control; ^#^P<0.05 versus HG.

There is increasing evidence that the mTOR (mammalian target of rapamycin) pathway is involved in the pathogenic manifestations of diabetic nephropathy. However, the mechanism by which mTOR promotes proximal tubular epithelial cell injury is unknown. mTORC1 is rapamycin sensitive and is thought to mediate many of its downstream effects through p70S6kinase/S6kinase 1 (S6K1) and 4E-binding protein 1 (4E-BP1). Rat proximal tubular cells were exposed to HG or NG for 48 hours in the absence or presence of 10 nm of rapamycin. Exposure of cells to HG for 48h significantly increases hypertrophy (Fig. 5*A*). This observation was associated with the activation of the mTORC1 pathway as determined by the phosphorylation of S6kinase on Thr^389^ (Figs. 5*A*–5*B*). Phosphorylation of mTOR^Ser2448^ was also increased (Figs. 5*A*–5*B*), an S6 kinase phosphorylation site. The deleterious effect of HG on tubular epithelial cells was reversed by the use of rapamycin (Fig. 5*A*). To determine if alteration in CYPs 4A and 2C regulates the activation of mTORC1 pathway; Rat proximal tubular cells were incubated with HG for 48 h in the absence or presence of HET0016 or MSPPOH. Treatment with HET0016 prevented HG-induced phosphorylation of S6kinase^Thr389^ as well as phosphorylation of mTOR^Ser2448^ (Fig. 5*B and 5D*). In contrast, treatment with MSPPOH stimulated the effect of HG-induced phosphorylation of S6kinase^Thr389^ as well as phosphorylation of mTOR^Ser2448^ (Fig. 5*C and 5E*). To our knowledge, these data and for the first time show that alteration in CYPs 4A/20HETE and CYPs 2C/EETs regulate the mTOR pathway playing a major role in diabetes induced tubulointerstitial changes and tubular injury.

## Discussion

Proximal tubular epithelial cell hypertrophy is a feature of diabetic nephropathy (DN) progression that contributes to tubular atrophy and thus leads to the loss of renal function. Oxidative stress has been implicated in the pathogenesis of diabetic complications including proximal tubular cell injury [40]. In this study, we demonstrate that DN is associated with a significant alteration in the expression and activity of cytochromes P450 and their AA metabolites, 20-HETE and EETs. Our data show that HG induces tubulointerstitial changes and tubular hypertrophy; effects mediated through the generation of ROS. Interestingly, we also demonstrate, that 20-HETE, produced by CYP4A, stimulate ROS production, increase extracellular matrix protein expression, and induce hypertrophy, an effect reversed by the use of HET0016 at short-term and long-term exposure to HG. In contrast, we show that long-term exposure of cells to HG (48 h of exposure) reduces CYP2C11 protein expression and EETs formation. This was paralleled by stimulation of ROS production, increase in extracellular matrix protein expression, and cellular hypertrophy. More interestingly, the use of MSPPOH, in the hyperglycemic milieu, induces tubulointerstitial changes and increases tubular injury. In this study, we also provide the first evidence about the crosstalk of cytochromes P450 with signaling proteins known to play a major role in cellular injury. Our data show that alteration in CYPs4A/20-HETE and CYPs2C/EETs regulate/activate the mTOR/S6 Kinase pathway, known to play an important role in the development of DN.

It has been well documented that diabetes induces oxidative stress and increases, in particular, the susceptibility of proximal tubular epithelial cells to oxidative damage leading to tubulointerstitial changes and tubular injury [40]–[48]. Blocking ROS production in the proximal tubules reduces many of the diabetic complications seen in the tubules [45]. But, the molecular mechanisms of action of oxidative stress as well as the precise sources of ROS have not been fully characterized. Mitochondrial electron transport chain, NADPH oxidases, and CYP450 are considered as potential sources of ROS in cells and tissues [48]. In this study, we show that CYP4A protein expression and 20-HETE formation were increased in cells exposed to HG at 6 h and 48 h (short and long-term), and that HG-induced ROS generation, matrix protein accumulation and proximal tubular cells hypertrophy were inhibited by HET0016, the potent inhibitor of 20-HETE production, suggesting that CYP4A-dependent 20-HETE formation induce tubulointerstitial changes and proximal tubular cells hypertrophy via enhanced ROS generation. In line with our work, CYP4A-dependent 20-HETE is highly produced in proximal tubules and exerts a wide range of regulatory and opposing functions depending on the location of its production [49]–[50]. Recent studies have identified oxidative stress as the mediator of the deleterious effects of 20-HETE. Zeng et al. (2010) showed that 20-HETE contributes to the myocardial injury during ischemia-reperfusion in cardiac myocytes [50]. Also, Eid et al. showed that inhibition of 20-HETE reverse, to a certain extent glomerular injury and albuminuria in diabetic mice by decreasing ROS production [22]. In contrast, in another study, 20-HETE has been described to have a renoprotective effect on the glomerular permeability barrier and may, thus slow the progression of matrix protein accumulation and renal fibrosis [13]–[15], [51]. These controversial findings show that the debatable role of 20-HETE still need further study, especially in identifying its crosstalk with signaling pathways known to play an important role in kidney injury.

What about the role of EETs? As for 20-HETE, EETs have been described to regulate cellular injury in a tissue- and disease-specific manner. EETs were shown to inhibit fibroblast proliferation and to promote apoptosis on platelet-derived growth factor (PDGF)-stimulated fibroblasts [52]. In contrast, EETs is also shown to inhibit apoptosis in carcinoma cells treated with arsenic trioxide [53]. In the kidneys, EETs has been shown to play a key role in cellular injury. Cisplatin-induced renal injury is significantly attenuated upon inhibition of soluble epoxide hydrolase (sEH), an enzyme that rapidly metabolizes EETs in the kidneys [54]. Moreover, genetic disruption of sEH in STZ-induced diabetic mice show significant decreased levels of creatinine, BUN and urinary microalbumin excretion. Also, sEH-deficient diabetic mice showed reduced kidney injury [55]. These data indicate a role for EETs in the biology of proximal tubular epithelial cells. However the mechanism of action and the metabolic pathway that can be regulated by EETs are still to be elucidated. In this study, we established that inhibition of EETs induces ROS production and mediates the deleterious effect of HG on proximal tubular cells by increasing fibronectin and collagen IV protein expression and by inducing cellular hypertrophy. In fact, we show that inhibition of EETs production by MSPPOH worsen the effect of HG-induced tubulointerstitial changes and proximal tubular epithelial cells injury assessed by increased hypertrophy. Therefore, we demonstrate that blockade of EETs after exposure to HG concentration is associated with increased ROS generation, which may account for the resulting proximal tubular cell injury in the hyperglycemic milieu. In support with our data, EET overproduction has been shown to inhibit renal tubular epithelial-mesenchymal transition, which sets the stage for renal fibrosis [56]. More interestingly, our data show that when cells are exposed, for short period, to HG there is an increase in both CYPs 4A and CYPs 2C protein expression as well as 20-HETE and EET formation. These observations are altered when cells are exposed for long-periods of time to HG, where CYPs 4A/20-HETE formation continue to increase, while CYPs 2C/EETs formation decrease dramatically, even to lever lower than those seen in cell exposed to NG. To our knowledge, our results are the first to describe that alteration in cytochromes P450 expression and their corresponding metabolites formation during the onset of the disease is different from the alteration of cytochromes P450 and their corresponding metabolites during the progression of the disease. Our data suggest an opposing effect of 20-HETE and EETs where increased levels of 20-HETE appear to be injurious to the kidney while increased EETs levels are supposed to be renoprotective.

In addition to the role of cytochromes P450 and their corresponding metabolite in inducing ROS production and tubular injury in diabetic nephropathy, we investigated the crosstalk of these enzymes and their metabolites with other signaling pathway involved in the development of DN are unknown. mTOR elicits a number of biological responses. In the kidney cortex of diabetic animals, the mTORC1/p70S6Kinase pathway is activated contributing to matrix accumulation [57]–[59]. In this study, we demonstrate that long-term exposure of proximal tubular cells to HG is associated with enhanced phosphorylation/activation of mTOR and its downstream effector p70S6kinase and induces cells hypertrophy. Our finding that rapamycin treatment inhibits HG-induced rat proximal tubular cells hypertrophy and apoptosis implicates mTOR in the pathogenic process of the progression of cells injury. This study also examined potential mechanism by which glucose enhances the phosphorylation and activation of mTOR in proximal tubular epithelial cells. We have demonstrated that excess glucose *in vitro* and after 48 h of incubation (long-term) results in the activation of CYPs 4A and induces 20-HETE production while it inactivates CYPs 2C11 and decreases EETs production. To our knowledge, this is the first study to show, that alteration in cytochromes P450 expression and their metabolites formation regulates the activation of the mTORC1/p70S6Kinase pathway. Importantly, these events correlate with proximal tubular epithelial cells injury observed in the hyperglycemic milieu.

On another note, a sex difference exists in susceptibility to diabetic kidney disease, with women showing protection against the development and progression of the disease compared with men. Responsibility for the observed differences has been attributed to sex hormones [60]. Studies in human beings, animal models, and renal cell culture have provided evidence in support of this hypothesis. However, the mechanisms by which sex hormones control the onset and development of diabetic nephropathy in both men and women remain to be elucidated. In rats, enzymes of the CYP450 4A and 2C families exhibit sexual dimorphism [61], but the physiological significance of this observation remains unknown. To our knowledge, studies in the literature that correlates sex difference, CYPs and diseases discuss cardiovascular dysfunction and blood pressure mechanisms. Experimental models and pathophysiological studies in humans suggest that enzymes involved in 20-HETE and EETs biosynthesis and metabolism have a role in the control of cardiovascular disease and blood pressure with a sex-specific effect. The results of several studies on different rat strains, using knockout models or specific pharmacological tools to alter the CYPs system, show a clear dimorphism between male and female animals: only male animals have an increased susceptibility to hypertension and either castration or the administration of androgen inhibitors revert the hypertensive phenotype [12], [62]–[66]. These observations may correlate hypothetically with the renoprotective effect seen in female diabetic animals where CYP 4A isoforms are expressed at high levels in male animals but not in females. However the physiological significance of these observations remains unknown in diabetes-induced kidney complications.

In conclusion, we have identified 20-HETE as a mediator of proximal tubular cell injury in the diabetic milieu while EETs may play a renoprotective effect in diabetic nephropathy. These results suggest that CYP4A inhibitors or EETs inducers may be useful in designing effective therapies, in addition to the metabolic control drugs, in the treatment of DN.
